# Lipidomics-Based Analysis of the Regulatory Effects of Phytosterol Esters on Lactation Performance and Lipid Metabolism in Tarim *Bactrian camels*

**DOI:** 10.3390/ani15192827

**Published:** 2025-09-28

**Authors:** Penglan Dou, Yusong Shen, Weihua Zheng, Lin Zhu, Yong Chen, Fengming Li

**Affiliations:** 1 College of Animal Science, Xinjiang Agricultural University, Urumqi 830052, China; dpenglan@163.com (P.D.); 18609937673@163.com (Y.S.); 320222603@xjau.edu.cn (L.Z.); xjaucy@163.com (Y.C.); 2 Xinjiang Academy of Agricultural Sciences, Urumqi 830091, China; zwhbachu@163.com

**Keywords:** plant sterol esters (PSEs), Tarim *Bactrian camel*, lipidomics, serum metabolites, milk metabolites

## Abstract

Plantsterol esters (PSEs) exert beneficial effects on animal product quality, indicating their potential as a nutritional intervention strategy. This study investigated the effects of dietary PSE supplementation on lactation performance and lipid metabolism in Tarim *Bactrian camels* (*Camelus bactrianus*) to establish a scientific basis for its application in their husbandry. Thirty-two mid-lactation female camels were randomly allocated to four groups (n = 8): CON (basal diet), L (200 mg/kg PSE), M (400 mg/kg PSE), and H (800 mg/kg PSE). Since lactation performance is closely linked to metabolic status, biochemical and lipidomic analyses were conducted on serum and milk samples. Analysis revealed that the H group showed significantly increased milk yield, lactose yield, and milk fat yield compared to other groups. Serum cholesterol levels decreased progressively with higher PSE supplementation, while serum urea levels rose dose-dependently. Blood Glu was lower in the L group but higher in the H group relative to CON. Lipidomic profiling identified 644 and 257 differential metabolites in milk and serum, respectively. Milk metabolites were enriched in the EGFR inhibitor resistance, MAPK, and ErbB signaling pathways; serum metabolites were linked to glycerophospholipid, arachidonic acid, and linoleic acid metabolism. These findings indicate that PSE-modulated metabolites in serum and milk significantly influence lactation performance and glucolipid metabolism in Tarim *Bactrian camels*, supporting further investigation into precision nutrition strategies.

## Simple Summary

In this study, dietary supplementation with phytosterol esters (PSEs) enhanced lactational performance and regulated lipid metabolism. To further explore the underlying mechanisms, lipidomic analyses of serum and milk were conducted. The results demonstrated that PSE significantly increased milk yield and the content of milk components, while decreasing serum cholesterol levels. Lipidomic profiling identified notable changes in lipid metabolites, including phosphatidylcholine and lysophosphatidylcholine, in both serum and milk. These metabolites were enriched in key pathways related to glycerophospholipid metabolism and linoleic acid metabolism, thereby contributing to optimized lipid metabolism and improved lactation performance. These findings provide a theoretical basis for the nutritional management of *Bactrian camels* and align with the existing literature on the cholesterol-lowering properties of PSE.

## 1. Introduction

The application of plant-derived bioactive compounds, collectively referred to as plant extracts, in animal production systems has attracted growing research interest in recent years. These natural extracts are increasingly regarded as promising alternatives to synthetic additives due to their well-established efficacy in enhancing livestock performance [1], health [2], and welfare [3,4]. They positively influence animal physiological functions through modulation of key metabolic pathways, immune responses, and overall homeostasis. Among various phytogenic active compounds, phytosterols (PS) have been extensively studied for their well-characterized roles in regulating lipid metabolism and immune function. PS are a class of bioactive compounds characterized by a cyclopentanoperhydrophenanthrene core structure and possessing antioxidant properties. As naturally occurring constituents predominantly found in plants, especially oilseeds, PS compete with cholesterol for incorporation into intestinal mixed micelles due to structural similarity, thereby inhibiting intestinal cholesterol absorption and/or modulating its metabolic pathways within animals [5]. Additionally, PS exhibit antioxidant, phytoestrogenic, and growth-promoting properties, and effectively modulate disruptions in systemic redox homeostasis [6]. In vitro studies have demonstrated that dietary phytosterol supplementation at 30 mg/kg enhanced nutrient digestibility by rumen microbiota and increased microbial protein synthesis in dairy cows [7]. Lv et al. [8] further reported that daily supplementation of 200 mg/d PS increased milk yield by 1.71 kg/d and reduced blood cholesterol levels in dairy cows. However, the inherent low bioavailability, suboptimal amphiphilicity (evidenced by a low average dissolved mass fraction of merely 3.0% to 5.0%), and susceptibility to degradation by light, heat, and oxidation collectively constrain PS applicability in certain contexts [9]. Phytosterol esters (PSEs), synthesized via esterification of PS with fatty acids, represent a novel functional feed additive. Compared to free sterols (PS), PSE are more readily absorbed by the animal intestine [10], exhibit improved fat solubility, achieve higher absorption and utilization [11], more effectively inhibit cholesterol absorption [11], and reduce sterol oxidation rates [12,13]. Current research has predominantly focused on dairy cattle, sheep, and monogastric species [14,15], while camels, a species exhibiting unique metabolic adaptations, remain underexplored.

The nutritional value of camel milk is intrinsically linked to the distinctive regulatory mechanisms governing lactation metabolism. Throughout lactation, significant metabolic alterations occur alongside dynamic changes in milk composition [16]. Serum metabolites critically participate in milk synthesis, serving as direct precursors for milk constituents and forming the physiological basis for converting dietary nutrients into milk components within the mammary gland [17]. In the mammary gland, Glu, amino acids, fatty acids, and other milk precursors from circulation enter mammary epithelial cells via specific transporters (e.g., amino acid and Glu transporters). These precursors are subsequently utilized to synthesize lactose, milk proteins, milk fat, and other nutrients [18,19]. The taste of camel milk varies depending on the feed and the availability of drinking water for the camels. Generally speaking, Camel milk appears white and opaque, with a slightly salty taste [20]. It exhibits relatively fat content (1.2~5.4%) [21], predominantly comprising polyunsaturated fatty acids (PUFAs) [22], and contains ~30 mg/100 g cholesterol (dry matter basis) [23]. Camel milk fat contains lower amounts of short-chain fatty acids and a higher proportion of long-chain fatty acids (C16-C18) as compared to buffalo and bovine milk [24]. It is particularly rich in vitamins B_1_, B_2_, and C [25]. Muthukumaran et al. [26] reported lower vitamins A and E but higher vitamin C (niacin) versus bovine milk, with vitamin C content approximately triple. This composition underpins its dietary significance in arid regions [27]. Furthermore, camel milk possesses beneficial nutritional and therapeutic properties—including antimicrobial, anti-inflammatory, antioxidant, and potential anticancer activities [28]—establishing it as a key resource for functional dairy product development. Phytosterol ester (PSE) degradation requires hydrolysis of sterol-fatty acid ester bonds by microbial esterases. As tylopoda, camels likely host uniquely adapted microbial esterase systems in their fore-stomachs. In contrast, camels are classified under the suborder Tylopoda and are distinguished by likely hosting uniquely adapted microbial esterase systems in their fore-stomachs, a feature markedly distinct from that of Ruminantia. Nevertheless, PSE metabolism in camels remains unexplored.

Metabolomics employs high-throughput technologies to detect endogenous metabolites in biological samples, offering significant advantages such as high resolution and sensitivity compared to conventional approaches [29]. As a major subfield of metabolomics, lipidomics utilizes advanced analytical techniques, particularly high-resolution mass spectrometry, to achieve systematic characterization of lipid molecular composition, accurate quantification, and elucidation of metabolic pathways across diverse sample types, including milk, blood, and meat [30]. Isa Fusaro et al. [31] applied lipidomics to compare sperm membrane lipid profiles between donkeys supplemented with hemp oil and a control group. Their findings revealed a marked increase in Eicosapentaenoic Acid (EPA), which is associated with omega-3 polyunsaturated fatty acid (PUFA) metabolism, along with enhanced membrane oxidative stability and a significant reduction in the peroxidation index in the supplemented group. This study demonstrates the utility of lipidomics in uncovering intricate lipid metabolic mechanisms.

While the individual functions of PSE and camel milk have been relatively well-studied, a significant research gap persists concerning camel metabolic responses to PSE intervention and blood–milk metabolic interplay. Therefore, this study aimed to investigate metabolic alterations in serum and milk of Tarim *Bactrian camels* (Camelus bactrianus) under graded PSE supplementation (0–800 mg/kg) using integrated metabolomics and conventional nutritional analysis, and examine effects on lactation performance, serum biochemical parameters, and serum-milk metabolite profiles. By elucidating intervention-induced metabolic changes, we aim to provide insights for optimizing nutritional management, enhancing milk yield, and improving Tarim *Bactrian camel* health.

## 2. Materials and Methods

### 2.1. Animals and Experimental Design

The animal experimental procedures were approved by the Animal Ethics Committee of Xinjiang Agricultural University (No: XJND2024018). Phytosterol esters (containing 31.13% total sterols, 50.79% sterol esters, and 0.12% free sterols) were purchased from Shanghai Zhongfeng Biotechnology Co., Ltd., Shanghai, China. The Tarim *Bactrian camels* in this study were obtained from the camel farm in Keping County, Aksu. Thirty-two healthy, mid-lactation (90–150 days) Tarim *Bactrian camels*, with an average weight of 434.44 ± 14.03 kg (mean ± standard deviation), an average age of 9.11 ± 1.05 years, and an average parity of 3.78 ± 0.83, were selected for this study. The camels were randomly assigned to four groups (n = 8): the control (CON) group, which was fed a basal diet; the L group, which received the basal diet supplemented with PSE (200 mg/kg); the M group, which was fed the basal diet supplemented with PSE at 400 mg/kg; and the H group, which received the basal diet supplemented with PSE at 800 mg/kg. The experiment included a 7-day adaptation period followed by a 35-day formal trial, with ad libitum water access. Milk yield data were recorded twice daily at 07:30 and 19:30. Camel milk samples were collected twice daily at 07:30 and 19:30 from each camel on days 0, 10, 30, and 35. The basal diet consisted of roughage feeds (rapeseed straw and silage) offered at a mass ratio of 1:2, mechanically processed to 3–4 cm particle length, with a dietary concentrate-to-roughage ratio maintained at 1:2.98 throughout the trial. The composition and nutritional level of refined materials are shown in Table S1.

### 2.2. Sample Collection and Analysis

Blood samples were collected from the jugular vein using 0.7 × 25 TW LB needles before morning feeding on days 1 and 35 of the trial period. After standing for 30 min, the blood samples were centrifuged at 3500 rpm for 10 min to separate the serum. The upper 2 mL of serum was collected from each tube and divided into three portions, which were then immediately frozen in liquid nitrogen and stored at −80 °C for subsequent analysis of serum biochemical parameters and metabolomics. Serum samples from Groups C to H are designated as BC, BL, BM, and BH, respectively. Serum biochemical parameters—including glucose (GLU), albumin (ALB), total protein (TP), triglycerides (TG), high-density lipoprotein cholesterol (HDL-C), low-density lipoprotein cholesterol (LDL-C), urea, and uric acid (UA)—were quantified using an automated biochemical analyzer (BS-240VET Mindray, Shenzhen, China).

Camel milk samples were collected from each camel at 07:30 and 19:30 on days 0, 10, 30, and 35, with a volume of 50 mL obtained per sampling. The samples were pooled in a 3:2 ratio (based on the morning-to-evening milk yield recorded daily during the trial), stored at 4 °C, and subsequently analyzed for milk composition using chemical methods. Determinations included moisture content (Direct drying method, GB 5009.3-2016), protein content (Kjeldahl method, GB 5009.5-2016), fat content (Gerber method, GB 5009.6-2016), and lactose content (Lane–Eynon method, GB 5009.8-2016) et al. [32,33,34,35,36]. Additionally, on the final morning of the experiment, extra samples were collected, snap-frozen in liquid nitrogen, and stored at −80 °C for further lipidomics analysis.

### 2.3. Lipidomic Analysis by UHPLC-MS/MS

UHPLC-MS/MS analyses were performed using a Vanquish UHPLC system (Thermo Fisher, Bremen, Germany) coupled with an Orbitrap Q ExactiveTM HF mass spectrometer (Thermo Fisher, Bremen, Germany) in Novogene Co., Ltd. (Beijing, China). An equal amount of supernatant was taken from each processed sample and mixed as a QC sample, which was used to monitor the deviation of the mixture and the analytical results of the analytical instrument itself. Samples were injected onto a ThermoAccucore C30 column (150 × 2.1 mm, 2.6 um) using a 20 min linear gradient at a flow rate of 0.35 mL/min. The column temperature was set at 40 °C. Mobile phase buffer A was acetonitrile/water (6/4) with 10 mM ammonium acetate and 0.1% formic acid, whereas buffer B was acetonitrile/isopropanol (1/9) with 10 mM ammonium acetate and 0.1% formic acid. The solvent gradient was set as follows: 30% B, initial; 30% B, 2 min; 43% B, 5 min; 55% B, 5.1 min; 70% B, 11 min; 99% B, 16 min; 30% B, 18.1 min. Q ExactiveT HF mass spectrometer was operated in positive [negative] polarity mode with sheath gas: 40 psi, sweep gas: 0 L/min, auxiliary gasrate: 10 L/min [7 L/min], spray voltage: 3.5 kV, capillary temperature: 320 °C, heater temperature: 350 °C, S-LensRF level: 50, scan range: 114–1700 *m*/*z*, automatic gain control target: 3 × 10^6^, normalized collisionenergy: 22 eV; 24 eV; 28 eV [22 eV; 24 eV; 28 eV], Injection time: 100 ms, Isolation window: lm/z, automatic gaincontrol target (MS2): 2 × 10^5^, dynamic exclusion: 6 s.

### 2.4. Lipidomics Data Processing

The raw offline data was initially imported into the LipidSearch database search software (version 5.1.12). Parameters such as a parent ion mass deviation of 5 ppm and a product ion mass deviation of 5 ppm are utilized to match the data with the LipidSearch database, facilitating peak extraction and the acquisition of qualitative substance information. Subsequently, peak alignment across samples is conducted using parameters including a retention time deviation of 0.05 min and a signal-to-noise ratio of 3. For clustering heat maps, the data were normalized using z-scores of the intensity areas of differential metabolites and were plotted using the Pheatmap package in R language. For multivariate statistical analysis, the data is processed using the metaX software (version R-3.4.3). Principal Component Analysis (PCA) and Orthogonal Partial Least Squares Discriminant Analysis (OPLS-DA) are then carried out using SIMCA (version 14.1) software to determine the Variable Importance in Projection (VIP) values for each metabolite. In the univariate analysis section, the statistical significance (*p* value) of each metabolite between the two groups is computed through a t-test, along with the calculation of the fold change (FC value) of metabolites between the groups. The default criteria for screening differential metabolites are set at VIP > 1, *p* value < 0.05, and FC ≥ 2 or FC ≤ 0.5. The KEGG database was used to study the functions and metabolic pathways of differential metabolites, and *p* < 0.05 was used as the criterion for significant enrichment. Venn diagrams were performed using the OmicStudio tools at https://www.omicstudio.cn (accessed on 9 July 2025). Additionally, using Spearman correlation analysis from [https://cloud.metware.cn] (accessed on 21 August 2025), the association between shared metabolites in serum and camel milk was evaluated. Furthermore, the Mantel test and Pearson correlation analysis were performed to assess the correlation between the composition of differentially expressed metabolites detected in serum and milk.

### 2.5. Statistical Analysis

The preliminary data processing was conducted using Excel 2019. Subsequently, one-way ANOVA and LSD multiple comparisons were conducted utilizing the ANOVA module of SPSS 26. software, encompassing both linear and quadratic analyses. The results were presented as mean values, standard errors (SEM), and *p*-values, denoted as *p* < 0.01 denotes a highly statistically significant difference, *p* < 0.05 indicates significant differences, while *p* > 0.05 indicates no significant differences.

## 3. Results

### 3.1. Effect of PSE on Lactation Performance

The lactation performance results of the C–H group are shown in Table 1. No significant differences were observed in milk protein yield, lactose yield, solids-not-fat (SNF) yield, or milk fat yield between groups L/M and the control group (*p* > 0.05). However, group H demonstrated optimal performance in key metrics, including milk yield, lactose, protein, fat, and SNF, suggesting potential multi-targeted regulation of milk component anabolism. Specifically, group H exhibited significantly higher milk fat yield (161.62 g/d; *p* < 0.01), lactose yield (188.15 g/d; *p* < 0.001), and average milk yield (+0.81 kg/d; *p* < 0.01) compared to the control. These results indicate that PSE intervention significantly enhanced milk yield, lactose, protein, fat, and SNF.

### 3.2. Effect of PSE on Serum Biochemical Parameters

The serum biochemical indices of the C–H group are presented in Table 2. Compared to the control group, the treatment groups L, M, and H exhibited lower levels of TC, HDL-C, and LDL-C. Notably, group M exhibited the lowest TC content, which was significantly lower than the control group (*p* < 0.05), but no significant differences were observed between group M and groups L and H (*p* > 0.05). Both groups M and H showed marginally lower LDL-C levels than the control (*p* = 0.051), although inter-group differences among treatments were non-significant, with a general trend of decreasing LDL-C as PSE supplementation increased.

Serum urea levels were significantly elevated in all treatment groups compared to the control (*p* < 0.01), with no differences among the groups. In contrast, serum Glu showed significant variations, with group H exhibiting the highest levels and group L the lowest (*p* < 0.05), indicating that the PSE intervention. Had differential effects on Glu metabolism. These results collectively suggest that moderate PSE supplementation effectively reduced serum TC and LDL-C levels, while simultaneously increasing serum urea content.

### 3.3. Lipid Metabolomics Multivariate Analysis of Serum and Camel Milk

**Figure 1:**
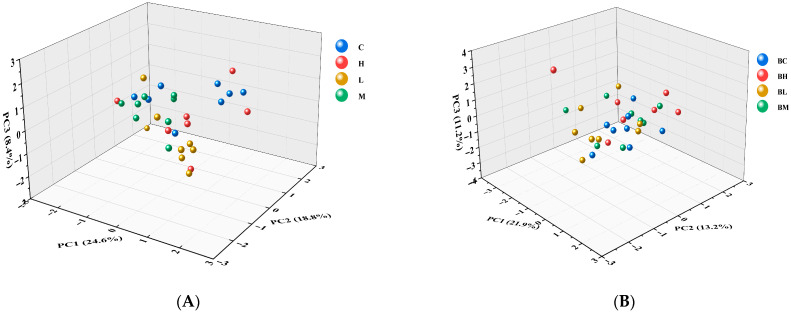
Multivariate statistical analysis of metabolites in different groups. (**A**) Principal component analysis PCA 3D score plot of camel milk samples based on lipidomics analysis (groups C, H, L, and M); (**B**) Principal component analysis PCA 3D score plot of serum samples based on lipidomics analysis (BC, BH, BL, and BM groups).

We performed multivariate statistical analysis and visualized the overall clustering pattern, demonstrating the distinctions between the serum and milk of four groups of Tarim *Bactrian camels* (Figure 1). The PCA 3D score plots of the camel’s milk samples revealed a distribution among groups (Figure 1A). Although there was some overlap between the four groups, a discernible pattern of metabolic changes from group C (control) to groups L, M, and subsequently H was apparent. The test and control groups showed a clear trend of separation along the PC1, PC2, and PC3 axes, indicating that the metabolite profiles of the test group significantly differed from those of the control group. Notably, the sample points of the control group (C) were relatively concentrated in the PCA plot, primarily distributed in the central region of PC1 and PC2 (blue points in the figure). This indicates that the metabolite differences among the samples of the control group are minimal and exhibit high similarity. Conversely, the sample points of group H (red points) are relatively distributed in the PCA plot were relatively dispersed in the negative regions of PC1 and PC2, which are significantly different from the control group.

The PCA 3D score plot of the serum samples (Figure 1B) revealed that the experimental treatments (BH, BL, and BM) induced significant metabolic alterations in the blood samples compared to the control (BC). These alterations are reflected in the apparent clustering and separation of the experimental and control groups. The metabolomic profile of group C and the L and M camels was almost completely covered, but all had distinct metabolomic clusters shared with group H. The metabolic profile of group C and the L and M camels was almost entirely overlapping, yet each group exhibited distinct metabolomic clusters that were shared with group H. The differences in the distribution of the test groups on the principal components indicated that each treatment had its unique metabolic characteristics.

To further highlight the differences between different treatment groups, OPLS-DA models were established using SIMCA-P software for metabolites in different groups, respectively. The 7-fold cross-validation, interpretability, predictability, and permutation test (n = 200) were used to measure the effectiveness of the model in OPLS-DA (Table 3). These models demonstrated good explanation rates and predictive abilities, indicating their reliability and robustness.

Meanwhile, the OPLS-DA score plot showed that the data from group C and other groups in camel’s milk were clearly separated, with significant differences in metabolic expression between the samples, confirming the model’s ability to effectively distinguish between groups and identify differential metabolites. Additionally, the permutation test plots for the L vs. C, M vs. C, and H vs. C groups indicated no overfitting as evidenced by the OPLS-DA substitution test, further supporting the model’s robustness. Similarly, the serum data from the BC group and other groups were also well-separated in the OPLS-DA score plot, with significant differences in metabolic profiles. The Permutation test results for the BL vs. BC, BM vs. BC, and BH vs. BC groups confirmed the absence of overfitting, indicating the stability and reliability of the OPLS-DA models.

### 3.4. Differential Lipid Metabolite Identification of Serum and Camel Milk

**Figure 2:**
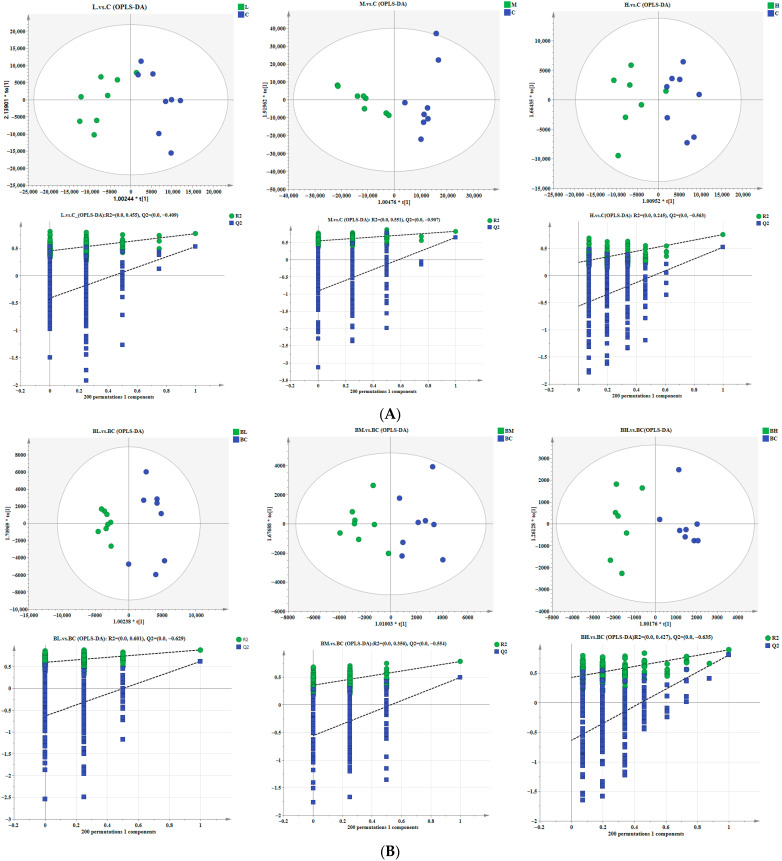
Orthogonal Partial Least Squares Discriminant Analysis (OPLS-DA) score plots, as well as permutation test plots for different groups of serum and camel milk samples. (**A**) OPLS-DA score plots and permutation test plots for camel milk samples; (**B**) OPLS-DA score plots and permutation test plots for serum samples.

**Figure 3:**
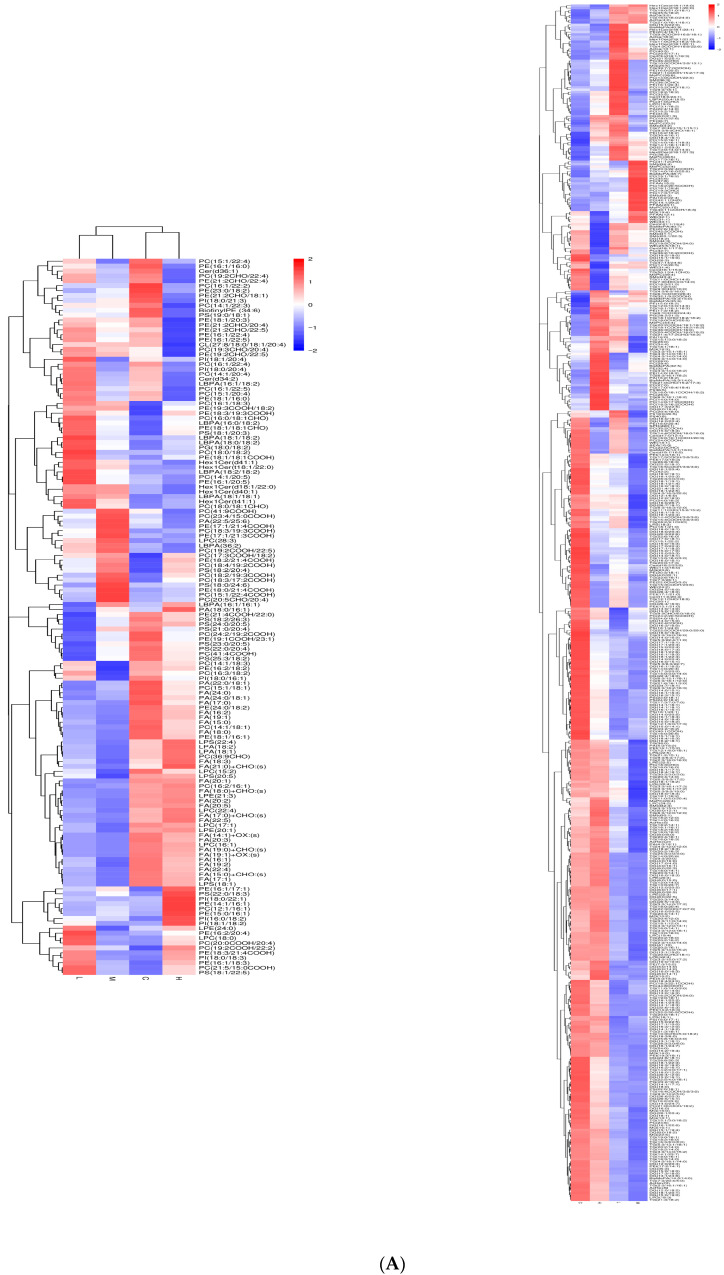

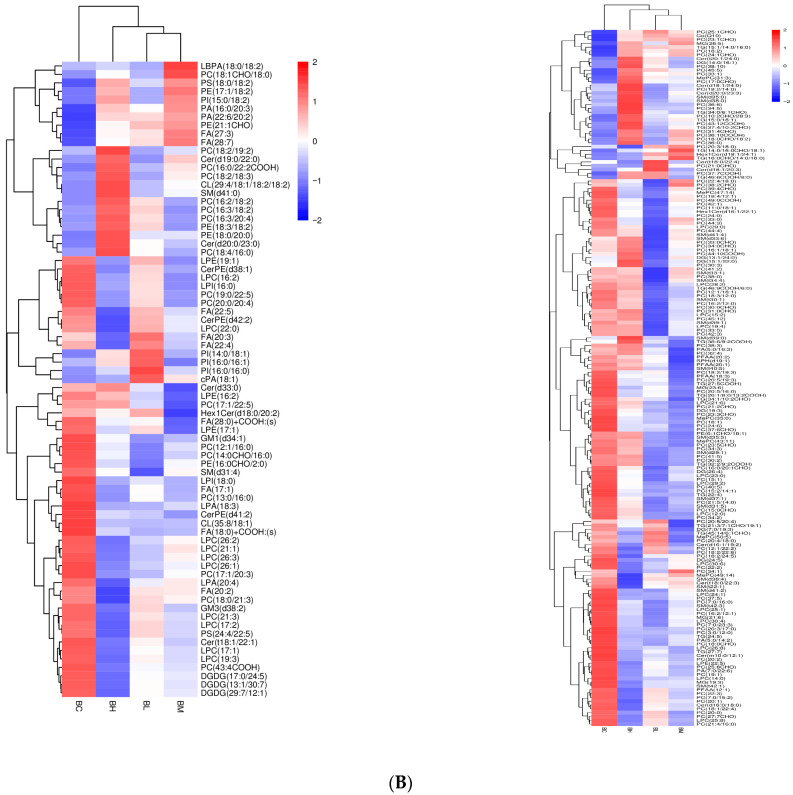
Cluster analysis of serum and camel milk samples. (**A**) Clustered heatmap of inter-group differentially expressed metabolites identified in camel milk groups C, L, M, and H (based on positive and negative ion modes). (**B**) Clustered heatmap of inter-group differentially expressed metabolites identified in serum groups BC, BL, BM, and BH (based on positive and negative ion modes).

**Figure 4:**
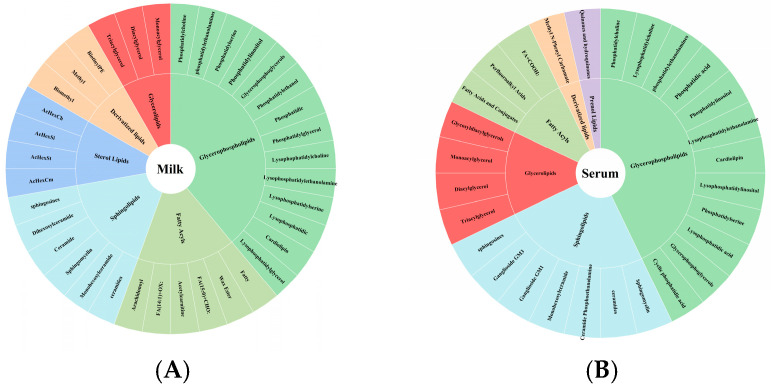
Classification of differential lipid metabolites in serum and camel milk samples. (**A**) Classification of camel milk differential metabolites. (**B**) Classification of serum differential metabolites.

The application of untargeted lipidomics analysis facilitated annotation of metabolites in both serum and camel milk, respectively (Figure 2A,B), and by combining lipid metabolites identified in both positive and negative ion modes, a total of 2732 metabolites were annotated in camel milk, while 2221 were annotated in serum. To identify differential metabolite distribution across groups, thresholds were set at VIP > 1.0, fold change (FC) > 1.2, or FC < 0.833 with *p*-value < 0.05. 644 differential lipid metabolites in camel milk and 257 in serum were identified, with the specific names and mass-to-charge ratios of these metabolites listed in Table S2. Cluster analysis was performed on differential lipid metabolites from both camel milk and serum. The heatmap visualization revealed distinct expression patterns across experimental groups, with a higher intensity of identification in camel’s milk in group H and the lowest in group M (Figure 3A); In Serum, the heatmap showed similar metabolic patterns in BH, BL, BM groups, while the BC group exhibited distinct metabolic profiles (Figure 3B). Classification of these differential metabolites by the Lipid search database indicated that camel milk metabolites consisted of glycerophospholipids, fatty acyls, sphingolipids, sterol lipids, derived esters, and glycerolipids (Figure 4A), whereas serum contained glycerophospholipids, fatty acyls, sphingolipids, derived esters, glycerolipids, and enol ketone lipids (Figure 4B).

### 3.5. Pathway Analysis of Serum and Camel Milk

**Figure 5:**
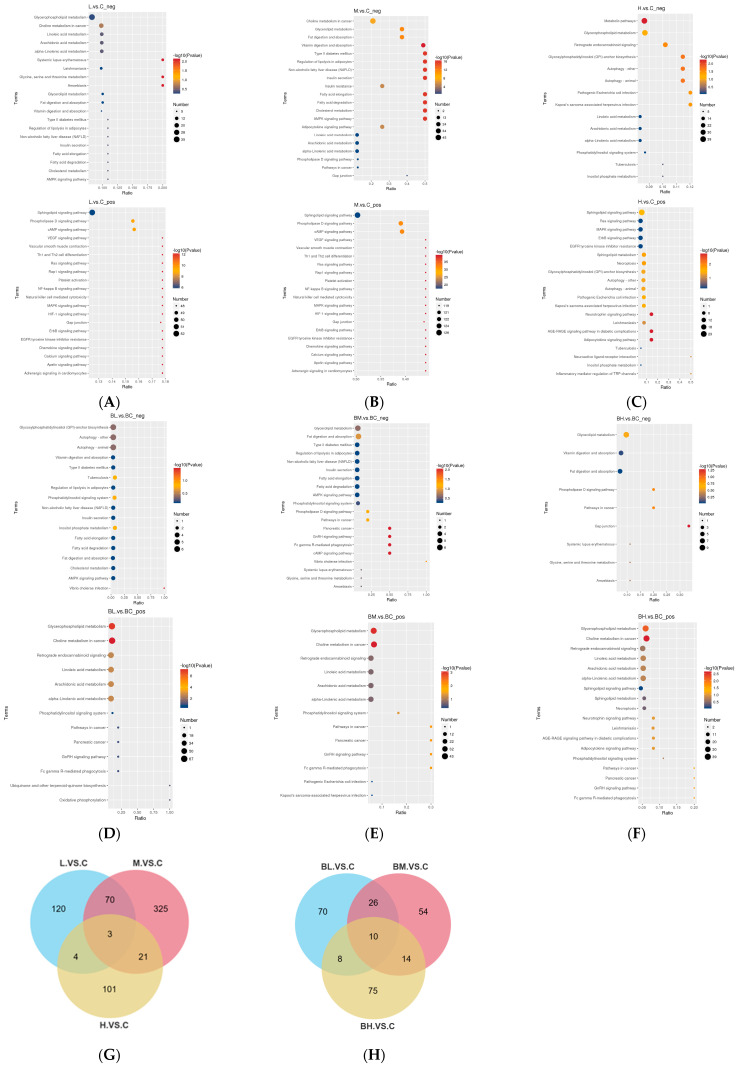
Functional pathway enrichment analysis of differential metabolites in pairwise comparisons of serum and milk samples. (**A**–**C**) Bubble chart analysis of functional pathways of camel’s milk from L vs. C, M vs. C, and H vs. C comparisons, respectively. (**D**–**F**) Bubble chart analysis of functional pathways of serum from L vs. C, M vs. C, and H vs. C comparisons, respectively. *X*-axis: x/y (number of differential lipid compounds in a pathway/total identified lipid compounds in that pathway), higher values indicate greater enrichment of differential lipids in the pathway. *Y*-axis: KEGG pathway names. The color and size of the bubbles indicate the *p*-value and the pathway impact index; the darker the bubble, the higher the *p*-value, and the larger the bubble, the higher the pathway impact index. (**G**,**H**) Venn diagrams of significantly different metabolites per experimental group.

To investigate associations between differential metabolites and metabolic pathways following PSE administration, functional pathway enrichment analysis was conducted on identified differential metabolites in serum and camel milk samples, and the top 20 pathways with the smallest *p*-value values were selected for visualization using bubble plots. For camel’s milk samples, the L vs. C group showed significant enrichment in pathways including Amoebiasis, Glycine metabolism, EGFR tyrosine kinase inhibitor resistance, and MAPK signaling pathway, related to immunity/inflammation, infection, metabolic regulation, and growth factor signaling (Figure 5A). The M vs. C group exhibited enrichment in AMPK signaling pathway, Insulin secretion, and Regulation of lipolysis in adipocytes, primarily involving metabolic regulation, particularly lipid and carbohydrate metabolism (Figure 5B). The H vs. C group was enriched in Metabolic pathways, Neurotrophin signaling pathway, and Adipocytokine signaling pathway, which are linked to metabolic regulation, neural processes, and diabetic complications (Figure 5C).

For serum samples, the BL vs. BC group exhibited significant enrichment in Glycerophospholipid metabolism and Choline metabolism in cancer (Figure 5D). The BM vs. BC group further expanded this enrichment by including additional pathways such as cAMP signaling pathway, Fc gamma R-mediated phagocytosis, and GnRH signaling pathway (Figure 5E). In contrast, the BH vs. BC group showed an even broader enrichment, encompassing Gap junction and Phospholipase D signaling pathway beyond aforementioned pathways (Figure 5F).

Integrated impact value analysis (*p*-value) revealed that serum metabolites were primarily enriched in Choline metabolism in cancer, Glycerophospholipid metabolism, Arachidonic acid metabolism, Linoleic acid metabolism, and alpha-Linolenic acid metabolism. In contrast, Camel milk metabolites showed primary enrichment in EGFR tyrosine kinase inhibitor resistance, MAPK signaling pathway, ErbB signaling pathway, Ras signaling pathway, Rap1 signaling pathway, and Sphingolipid signaling pathway. Combined positive and negative ion mode Wayne plots demonstrated that in camel’s milk, 197, 419, and 129 differentially altered metabolites were identified between the L vs. C, M vs. C, and H vs. C groups, respectively (Figure 5G). For serum, 114, 104, and 107 differential metabolites were detected in BL vs. BC, BM vs. BC, and BH vs. BC groups, respectively (Figure 5H), Among these, three shared metabolites were identified in camel’s milk: DG (16:0/16:1), TG (12:1CHO/18:0), and Cer (d18:2/23:0); while 10 serum-shared metabolites included serum-shared metabolites were CL (35:8/18:1), PE (21:1CHO), LPC (25:1), PC (3:0/12:0), PC (7:0/16:0), PC (20:2), PA (7:0/22:6), LPC (26:8), LPC (24:1), PA (5:0/14:2).

### 3.6. Interaction Analysis of Serum and Camel Milk Lipid Metabolites

**Figure 6:**
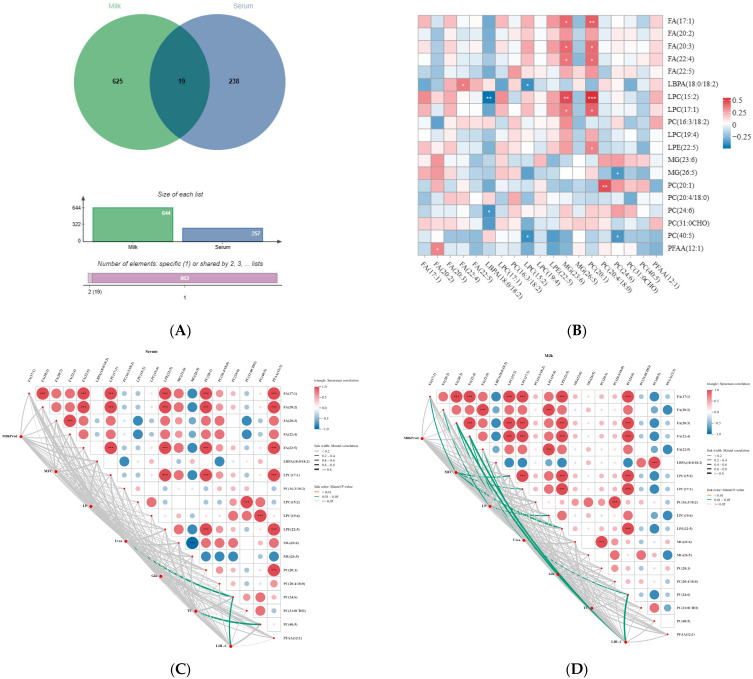
Identification of shared differential metabolites in serum and camel milk for analysis. (**A**) Venn diagram of the distribution of shared differential metabolites of serum and camel milk for C–H. Blue circles indicate serum, green circles indicate camel milk, and dark-colored circles indicate that serum is shared with camel milk. (**B**) Pearson correlation analysis of common differential metabolites in serum and camel milk of C–H groups. Horizontal coordinates indicate shared metabolites in camel milk, vertical coordinates indicate shared metabolites in serum, * represents 0.01 < *p* < 0.05, ** represents 0.001 < *p* < 0.01, and *** represents *p* < 0.001. (**C**,**D**) Correlation network analysis of shared differential metabolites with indicators of difference in serum and camel milk. (**C**) association of serum shared differential metabolites with biochemical indices, (**D**) association of camel milk shared differential metabolites with biochemical indices. Correlations between metabolites and differential indicators were determined using Mantel’s tests, with the thickness of the connecting line indicating the correlation coefficient; the color of the connecting line indicated significance, with green being a significant correlation (0.01 < *p* < 0.05). Pearson’s test tested correlations between shared differential metabolites; box size and color gradient indicate Pearson’s correlation, red indicates positive correlation, blue indicates negative correlation, * represents 0.01 < *p* < 0.05, ** represents 0.001 < *p* < 0.01, and *** represents *p* < 0.001. Abbreviations: MFC, Milk fat yield; LP, Lactose yield.

To further explore the correlation between the differential metabolites in the serum and milk, the 257 metabolites identified in the serum samples were compared with the 644 metabolites identified in the camel milk samples. It was found that 19 (2.15%) of the differential metabolites shared between the four groups of the milk and serum from 4 groups, with camel milk alone accounting for 625 (70.86%) and serum alone accounting for 238 (26.99%) of the differential metabolites (Figure 6A). Spearman’s correlation analysis of these 19 shared differential metabolites revealed that serum LPC (15:2) positively correlated with milk MG (23:6) and PC (20:1) (*p* < 0.05), but negatively correlated with LBPA (18:0/18:2). Serum PC (20:1) positively correlated with milk PC (20:4/18:0) (Figure 6B). At the same time, the physiological indices with significant differences in the 4 groups serum or camel milk were screened based on the previous results, and Mantel’s correlation was performed with 19 shared differential metabolites (Figure 6C,D). The main indicators with significant differences between the four groups were milk yield, milk fat, lactose, serum urea, serum Glu, total cholesterol, and LDL-C. Urea content was found to be significantly correlated with PC (24:6) (*p* < 0.05) in serum and also significantly correlated with PC (24:6) (*p* < 0.05) in camel’s milk; TC content in serum showed significant correlation with PC (40:5) (*p* < 0.05), and LDL-C content in serum showed significant correlation with PC (24. 6) (*p* < 0.05), which was also significantly correlated with FA (20:3) (*p* < 0.05), FA (22:4) (*p* < 0.05) in camel’s milk; milk fat (MFC) was significantly correlated with FA (17:1), LPC (15:2), LPC (17:1), LPC (19:4), LPE (22:5) in camel’s milk (*p* < 0.05); lactose (LP) was significantly correlated with FA (20:2) and LPC (19:4) in milk (*p* < 0.05).

## 4. Discussion

Camel milk is a nutritious natural food, but its quality is influenced by numerous factors, including breed, parity, lactation stage, season, nutritional level, and feeding management [37]. Milk yield, a key indicator for assessing the production efficiency of *Bactrian camels*, is affected by genetic factors, nutritional status, and physiological condition. Numerous studies have demonstrated that supplementation of PS can increase milk yield in dairy cows. Lv et al. [38] reported that adding 40 and 80 g/d PS to the diet of early-lactation Holstein cows increased milk yield by 2.07 and 1.74 kg/d, respectively. Jin et al. observed that dietary supplementation with 200 and 800 mg/d PS increased milk yield by 1.71 and 0.44 kg/d, respectively, in dairy cows [39]. The results of this experiment confirmed that PSE also increases milk yield, consistent with previous findings. Supplementation with 800 mg/kg PSE significantly increased milk yield in Tarim *Bactrian camels* (group H: 3.36 kg/d vs. group C: 2.76 kg/d), and the milk fat percentage increased from 2.19% in the control group to 4.81%. The increase in milk fat yield in the treatment group may be attributed to the cholesterol-lowering effect of PSE, leading to increased relative concentrations of triglycerides, phospholipids, and free fatty acids in serum lipids. Consequently, the uptake of fat by mammary gland acinar epithelial cells from the serum increased, thereby elevating milk fat yield. Alternatively, PSE may reduce serum LDL-C levels, promoting increases in VLDL and chylomicrons, which enhance triglyceride transport and fat synthesis, ultimately increasing milk fat yield.

Concurrently, significant variations in lactose yield were observed across supplementation levels, with the H group (800 mg/kg PSE) exhibiting the highest values. Lactose biosynthesis occurs within the Golgi apparatus of mammary epithelial cells, where the lactose synthase enzyme complex catalyzes the condensation of Glu and uridine diphosphate galactose (UDP-galactose) into lactose. As blood Glu constitutes the direct metabolic precursor for lactose production, the elevated blood Glu levels associated with increased PSE supplementation in the present study correspond mechanistically to the concurrent rise in milk lactose content observed experimentally. Incremental PSE dosage also elevated milk protein and other major components. One potential explanation is that PSE binds to molecular membranes upon entering the body, forming nucleoprotein complexes that stimulate animal protein synthesis [40]. Alternatively, PSE may exhibit estrogen-like activity, regulating physiological metabolism post-absorption, promoting anabolic processes, reducing catabolism, and eliciting corresponding physiological responses [41]. The precise mechanisms warrant further investigation.

Serum biochemical indices serve as vital indicators of physiological status, reflecting an organism’s growth, development, nutritional metabolism, and health [42]. Triglycerides (TG), total cholesterol (TC), and non-esterified fatty acids (NEFA) reflect lipid absorption and metabolism. PSEs are known to inhibit enzyme systems involved in cholesterol synthesis [43]. Consistent with prior findings in laying hens (where 20–40 mg/kg PSE reduced serum TC and LDL-C [44]), this study demonstrated that PSE significantly decreased serum TC (group M: 1.35 mmol/L vs. group C: 1.83 mmol/L) and LDL-C (group H: 1.10 mmol/L vs. group C: 1.55 mmol/L). Serum TC and LDL-C primarily reflect serum lipid metabolism. While studies report reduced serum TG in piglets supplemented with esterified PS [45], this experiment showed no significant TG differences. Serum GLU concentrations exhibited a significant quadratic response to increasing PSE supplementation, decreasing initially before rising at the highest dose. This biphasic pattern may be explained by the ability of moderate PSE supplementation to improve insulin sensitivity through reductions in circulating and tissue lipid accumulation, thereby promoting Glu uptake in skeletal muscle and adipose tissue and inhibiting hepatic gluconeogenesis, ultimately leading to decreased blood Glu levels [46]. However, studies indicate that excessively high doses of PS can alter biomarkers such as alkaline phosphatase (AKP), suggesting potential hepatic metabolic stress [37]. As the central regulator of Glu homeostasis, the liver may experience an increased metabolic burden under high PSE exposure, leading to mild hepatic stress. This could disrupt the balance between gluconeogenesis and glycogen synthesis/degradation [47], resulting in elevated blood Glu. The exact mechanisms underlying this effect require further investigation. Simultaneously, Glu, as the primary energy substrate of the organism, is also an important substance required for lactose synthesis. Notably, group L showed reduced TG (0.19 vs. 0.28 mmol/L), potentially attributable to low-dose PSE activating lipoprotein lipase (LPL) and promoting TG hydrolysis [11]. Unchanged TG in group H implies preferential redirection toward fatty acid synthesis at higher doses. Collectively, PSE supplementation in *Bactrian camels* lowers cholesterol, optimizes energy metabolism, and potentially improves health status.

Metabolomics technology is now extensively applied in nutritional science, toxicology, and drug development to elucidate molecular metabolic responses to nutrients, toxicants, and pharmaceuticals in humans and animals. PSEs ameliorate lipid metabolic disorders by modulating glycerophospholipid and ether lipid pathways, thereby reducing high-fat diet-induced lipid accumulation [48]. Lipids are indispensable cellular molecules critical for membrane integrity, energy storage, and signal transduction. Among 664 differential lipids identified in camel milk, triacylglycerols (TG, 166 species) constituted the predominant subclass, followed by diacylglycerols (DG, 144), phosphatidylcholines (PC, 91), and phosphatidylethanolamines (PE, 47). In serum (257 differential lipids), PC (108) was most abundant, followed by lysophosphatidylcholines (LPC, 25), sphingomyelins (SM, 21), and TG (18). As the primary neutral lipid subclass, TG features fatty acids (FAs) esterified at sn-1, sn-2, and sn-3 positions, serving as crucial energy reservoirs for cellular growth and metabolism [49]. TGs represent the main lipid storage form, with substantial FA incorporation [49], and exhibit trophic correlations between backbone-esterified FAs [50]. During energy mobilization, TG-derived FAs undergo hydrolysis, mitochondrial β-oxidation, and acetyl-CoA production, ultimately fueling energy generation via the tricarboxylic acid (TCA) cycle. This experiment revealed significant TG divergence between serum and camel milk, suggesting serum TG supplies substrates for de novo milk TG synthesis.

Diacylglycerol (DG), a signaling molecule regulating cellular activities, influences lipid metabolism and is widely utilized in the food industry [51]. As intermediates in milk lipid synthesis, DG-like metabolites containing arachidonic acid at the sn-2 position elevate triacylglycerol (TAG) accumulation in mammary epithelial cells by ~25% through enhanced DGAT1 enzyme activity [52], indirectly modulating milk lipid content. Phosphatidylcholine (PC)—essential for lipoprotein assembly/secretion and lipid distribution [53] —promotes lipid droplet formation when cellular PC content increases [54], consistent with the elevated milk fat yield observed in this experiment. Minor sphingolipids detected in serum and camel milk differential lipids included sphingomyelins (SM), hexosylceramides (Hex1Cer), and ceramides (Cer). Hex1Cer regulates cell adhesion, differentiation, proliferation, apoptosis, and stress responses [55], exerting pivotal anti-inflammatory and immunomodulatory functions. SM modulates lipid absorption and cholesterol efflux; dietary SM reduces hepatic/plasma lipids in obese/diabetic mice by inhibiting intestinal lipid absorption and promoting cholesterol excretion [56]. These mechanisms align with the reduced TG, TC, and LDL-C levels observed in our trial.

Based on lipidomics data, KEGG pathway enrichment analysis of differential lipids among groups revealed key metabolic pathways in serum, encompassing glycerophospholipid metabolism, arachidonic acid metabolism, linoleic acid metabolism, and alpha-linolenic acid metabolism. This indicates significant alterations in serum lipid pathways, correlating with changes in the lipid composition of camel milk. Glycerophospholipid metabolism in milk is closely associated with maternal health status; for example, maternal obesity or inflammatory conditions may disrupt milk phospholipid metabolism, modifying fatty acid composition and ratios [57]. In this trial, enriched metabolites within the glycerophospholipid metabolism pathway primarily comprised PC, LPC, and PA, indicating that enhanced glycerophospholipid metabolism is critical for lipid biosynthesis. Lysophosphatidylcholine (LPC) and acyl-CoA are catalyzed by phospholipase A_2_ to synthesize phosphatidylcholine (PC), whereas PC is cleaved by lysophospholipid acyltransferase to generate LPC [58]. This indicates that PSE intervention significantly enriches the glycerophospholipid metabolism pathway. Within this pathway, metabolites including LPC, PC, and phosphatidylserine (PS) critically regulate systemic lipid metabolism, thereby enhancing lactation performance.

To elucidate the relationship between serum and milk lipid molecules during PSE-mediated lipid metabolism regulation in Tarim *Bactrian camels*, integrated lipidomics analysis of serum and camel milk was conducted. Correlation analysis identified highly correlated metabolites: serum LPC (15:2) and PC (20:1) with camel milk MG (23:6), PC (20:1), LBPA (18:0/18:2), and PC (20:4/18:0). Among them, LPC associates with inflammatory diseases, is generated via phospholipase A2-mediated phosphatidylcholine hydrolysis, and modulates glycemia and insulin resistance [59]; PC constitutes > 50% of mammalian membrane phospholipids [60], participates in lipid/energy metabolism and neurotransmitter synthesis [61], and its dysregulation induces lipid disorders [62]; LBPA, either directly enriched or via its precursor phosphatidylglycerol (PG), has been investigated as an intervention to reduce cholesterol accumulation [63], affecting lipid homeostasis by regulating in vivo cholesterol transport. Correlation analysis of these metabolites with physiological indices revealed that milk fat percentage positively correlated with camel milk metabolites FA (17:1), LPC (15:2/17:1/19:4), and LPE (22:5). FA, a saturated fatty acid typically associated with milk fat synthesis, showed a positive correlation with milk fat percentage, indicating its important role in this process. Additionally, FA concentration may be influenced by rumen fermentation and fatty acid availability in feed [64], while the significant increase in milk fat yield following PSE supplementation suggests improved lipid metabolism and promotion of milk fat synthesis. Lactose is synthesized by mammary epithelial cells [65], with serum Glu serving as the primary precursor delivered to support its synthesis. This study observed higher serum Glu concentration and lactose yield in Group H. Results showed that lactose yield in Group H was significantly higher than that of the control group and correlated with FA (20:2) and LPC (19:4), indicating that lactose yield can be increased by regulating serum Glu levels. However, synergistic mechanisms between lactose, FA (20:2), and LPC (19:4) require further investigation.

This study provides initial evidence supporting a significant role for PSE intervention in modulating glycolipid metabolism in Tarim *Bactrian camels*. It should be noted, however, that the limited sample size may constrain the statistical power and generalizability of these findings. Furthermore, the differences observed throughout the experimental period may not solely represent inherent inter-breed variability and could be confounded by uncontrollable external factors. Therefore, larger-scale studies are required to confirm these results. Subsequent investigations should also aim to elucidate the mechanisms by which PSE-induced alterations in serum metabolite profiles influence milk production and composition, which will aid in developing targeted nutritional strategies. In addition, integrating metabolomics with other omics technologies, such as microbiomics, transcriptomics, and proteomics, will be crucial for a holistic understanding of the metabolic networks regulated by PSE.

## 5. Conclusions

This study elucidated that the addition of 800 mg/kg PSE significantly increased milk yield, milk fat yield, and milk lactose yield. PSE supplementation significantly lowered serum total cholesterol and low-density lipoprotein cholesterol levels, suggesting competitive inhibition of intestinal cholesterol absorption. Lipidomics revealed PSE induced significant alterations in serum and camel milk lipids and energy metabolites, with PC, LPC, and LBPA identified as key regulators. These findings delineate the relationship between serum and camel milk metabolomes and milk yield, underscoring the importance of meeting nutritional requirements. The exact mechanisms involved require further investigation to be elucidated.

## 6. Patents

The patent applicant for this work is Xinjiang Agricultural University and the Feed Research Institute of the Xinjiang Uygur Autonomous Region Academy of Animal Science. The names of inventors are Fengming Li, Penglan Dou, Kaixu Chen, Junyu Zhang, Jiancheng Liu, Yusong Shen, Junze Niu, and Di Wen. The application number is 202511029520X, and the status of the application is currently pending.

## Figures and Tables

**Table 1:** Absolute quality index and significance analysis of milk composition.

Items	C Group	L Group	M Group	H Group	SEM	Treatment	Linear	Quadratic
Milk yield (kg/d)	2.76 ^Bb^	2.76 ^Bb^	2.92 ^ABb^	3.57 ^Aa^	1.254	<0.01	<0.01	0.063
Lactose yield (g/d)	159.79 ^Bab^	154.65 ^Bb^	166.24 ^Bb^	188.15 ^Aa^	16.730	<0.001	<0.001	<0.001
Milk protein yield (g/d)	101.95 ^Bbc^	99.35 ^Bc^	105.94 ^Bb^	118.40 ^Aa^	10.281	<0.001	<0.001	<0.01
Milk fat yield (g/d)	60.49 ^Bb^	72.11 ^Bb^	78.09 ^Bb^	161.62 ^Aa^	53.396	<0.01	<0.01	0.135
Non-fat solids (g/d)	245.43 ^Bbc^	236.36 ^Bc^	254.72 ^Bb^	283.82 ^Aa^	26.163	<0.001	<0.001	<0.01

Note: In the same row, values with different small letter superscripts indicate a significant difference (*p* < 0.05); values with different capital letter superscripts indicate a highly significant difference (*p* < 0.01); while values with the same letter superscripts indicate no significant difference (*p* > 0.05). The following table is the same.

**Table 2:** Effect of PSE on Serum Biochemical Parameters.

Items	C Group	L Group	M Group	H Group	SEM	Treatment	Linear	Quadratic
Glu mmol/L	6.39 ^ABab^	5.75 ^Bb^	6.59 ^ABab^	7.03 ^Aa^	0.837	<0.05	<0.05	0.063
TP g/L	66.96	67.99	66.59	65.27	4.454	0.745	0.403	0.504
ALB g/L	43.50	42.80	43.77	43.47	3.776	0.972	0.893	0.894
GLB g/L	23.46	25.19	22.81	21.81	3.928	0.454	0.270	0.363
A/G	1.92	1.77	1.96	2.02	0.364	0.639	0.425	0.461
Urea mmol/L	8.02 ^Bb^	9.53 ^Aa^	9.76 ^Aa^	9.78 ^Aa^	1.091	<0.05	<0.001	0.051
UA μmol/L	13.40	16.17	13.86	18.33	6.050	0.501	0.296	0.738
TG mmol/L	0.28	0.19	0.28	0.27	0.090	0.197	0.715	0.228
TC mmol/L	1.83 ^Aa^	1.47 ^ABb^	1.35 ^Bb^	1.43 ^ABb^	0.336	<0.05	<0.05	0.055
HDL-C mmol/L	0.31	0.30	0.25	0.29	0.070	0.426	0.361	0.484
LDL-C mmol/L	1.55 ^a^	1.35 ^ab^	1.13 ^b^	1.10 ^b^	0.340	0.051	<0.05	0.500

Abbreviations: Glu, glucose; TP, total protein; ALB, albumin; GLB, globulin; A/G, ALB/GLB ratio; UA, uric acid; TG, triglycerides; TC, total cholesterol; HDL-C, high-density lipoprotein cholesterol; LDL-C, low-density lipoprotein cholesterol. In the same row, values with different small letter superscrpts indicate a significant difference (*p* < 0.05); values with different capital letter superscripts indicate a highly significant difference (*p* < 0.01); while values with the same letter superscripts indicate no significant difference (*p* > 0.05).

**Table 3:** Evaluation Parameters of the OPLS-DA Model.

Groups	A	N	R2X	R2Y	Q2
L vs. C	1 + 1	16	0.833	0.77	0.534
M vs. C	1 + 2	16	0.811	0.829	0.651
H vs. C	1 + 1	15	0.597	0.759	0.525
BL vs. BC	1 + 2	16	0.835	0.886	0.624
BM vs. BC	1 + 1	16	0.703	0.786	0.528
BH vs. BC	1 + 1	15	0.704	0.896	0.807

Note: A = number of components; N = sample size; R2X = explained variance in X-variables; R2Y = explained variance in Y-variables; Q2 = predictive ability.

## Data Availability

The original contributions presented in this study are included in the article/Supplementary Materials. The production data supporting this study cannot be shared publicly due to ongoing patent proceedings. They will be available from the corresponding author upon reasonable request after the patent is granted. Further inquiries can be directed to the corresponding author.

## Supplementary Materials

The following supporting information can be downloaded at https://www.mdpi.com/article/10.3390/ani15192827/s1 and https://data.mendeley.com/drafts/h2r57xn2tr (accessed on 24 September 2025), Table S1: Composition and nutrient levels of concentrate (DM basis) %; Table S2: Lipidomics-based analysis of the regulatory effects of phytosterol esters on lactation performance and lipid metabolism in Tarim Bactrian camels.

## Abbreviations

The following abbreviations are used in this manuscript:

PSE	Plant sterol esters
Glu	Glucose
TP	Total protein
ALB	Albumin
A/G	ALB/GLB ratio
UA	Uric acid
TG	Triglycerides
TC	Total cholesterol
HDLC	High-density lipoprotein cholesterol
LDLC	Low-density lipoprotein cholesterol
