# Supplementary material for: Lipidomics-Based Analysis of the Regulatory Effects of Phytosterol Esters on Lactation Performance and Lipid Metabolism in Tarim Bactrian camels
Source: Animals (Basel). 2025 Sep 28;15(19):2827. doi: 10.3390/ani15192827 (PMC12523444; doi:10.3390/ani15192827)
Supplement: Supplementary file 1 [file animals-15-02827-s001.zip › animas-3867755 (Table S1).pdf]

Table S1. Composition and nutrient levels of concentrate (DM basis) %.

Ingredients	Contents	Nutrient Levels [2]	Contents
Corn	63.04	OM	92.38
Wheat bran	9.7	CP	19.05
Soybean meal	19.4	Fat	3.58
Limestone	1.62	CF	5.71
Amino acid	1.62	Ca	0.56
NaCl	1.62	P	0.23
Premix [1]	1.08	GE（MJ/kg）	17.75
CaHPO4	1.94		
Total	100.00		

[1] Premix provides iron (ferrous sulfate) 135.0 mg, iodine (potassium iodide) 1.5 mg, manganese (manganese sulfate) 60.0 mg, zinc (zinc sulfate) 50.0 mg, copper (copper sulfate) 15.0 mg, vitamin A 3 600 IU, vitamin D 2800 IU, vitamin E 1 200 IU, vitamin B1 4.5 mg, vitamin B2 3.0 mg per kg of diet. [2] Nutrient levels are measured values.
